# Analysis of ballistic trajectories and its association with clinical outcomes in civilian penetrating brain injury

**DOI:** 10.1007/s00068-024-02643-3

**Published:** 2024-09-09

**Authors:** Sebastián Ordoñez, Mauricio A. Ledesma, Lina María Villegas-Trujillo, Miguel Velásquez, María Trujillo, Andrés M. Rubiano

**Affiliations:** 1 Neurosurgery Section, School of Medicine, Universidad del Valle, Street 5, Cali, #36-00 Colombia; 2 Hospital Universitario del Valle, Cali, Colombia; 3 Department of Natural and Exact Sciences, Universidad del Valle, Cali, Colombia; 4 School of Systems and Computing Engineering, Universidad del Valle, Cali, Colombia; 5 INUB-Meditech Research Group, Neuroscience Institute, Universidad El Bosque, Bogotá, Colombia

**Keywords:** Traumatic brain injury, ​​Penetrating brain injury, Ballistic trajectory, Vectorial analysis, Gunshots outcomes

## Abstract

**Purpose:**

Civilian penetrating brain injuries (PBI) caused by firearms are a medical emergency with high rates of morbidity and mortality. The aim of this study was to evaluate the association between trajectory vectors in CT brain angiography and clinical outcomes in patients with civilian gunshots.

**Methods:**

This is a retrospective analytical cross-sectional study that includes patients over 15 years of age with PBI due to firearms, admitted from January 2019 to December 2021 at a University Hospital in Cali, Colombia. A brain CT with angio-CT was performed the first day of admission. An XYZ coordinate system centered on the Turk’s saddle was developed. Trajectories of projectiles were plotted and compared to a patient 0 in a 3D-Slicer software. A bivariate analysis of the clinical and geometric characteristics of the trajectory was performed. Primary outcomes include mortality and disability at 6 months.

**Results:**

Twenty-eight patients with a mean age of 27.39 ± 11.66 years were included. The vectors of non-survivors show a trend, crossing at a specific area. This area was designated as a “*potential lethal zone*” and inside this area, injuries around 25.3 mm from the circle of Willis, were associated with greater mortality (*p* < 0.005).

**Conclusions:**

In our study PBI avoiding the ventricular system, brain stem, dorsum sellae and the circle of Willis were associated with more survivability. A “potential lethal zone” was detected and associated with poor outcome after civilian PBI due to firearms. A better evaluation of the performance of this “*potential lethal zone*” in larger studies will be required.

## Introduction

Civilian gunshots to the head refer to a specific type of penetrating brain injury (PBI) considered an emergency with high rates of mortality and morbidity [1]. It has been described that 10% of patients with these types of injuries survive and reach the hospital, half of them die in the emergency department and the other half often suffer severe long-term disabilities [2]. Mortality rates in these types of injuries has been described as high as 66 to 93% in some series and epidemiological studies [1, 2].

Knowing the predictors of favorable and unfavorable results based on the initial CT imaging is critical for surgeons who manage this type of conditions. The vector analysis of the bullet trajectory is considered one of these factors and has been critical in the correlation with patient’s outcomes [3, 4]. Specific penetrating and perforating types of PBI in CT scan are associated with higher mortality, such as bitemporal, multilobe and trans-ventricular injuries [3]. However, most of these imaging predictors have not been studied in CT brain angiography, which is an important advanced tool for evaluating vascular affections in penetrating traumatic brain injury (TBI).

The present study aimed to identify the association between the bullet trajectory, CT brain angiography vector analysis and clinical outcomes in patients with civilian PBI. The primary outcomes include mortality and disability at 6 months. Our hypothesis suggests that involvement of brain areas near to vascular structures can be associated with higher mortality.

## Materials and methods

### Collection of data and patient selection

**Fig. 1:**
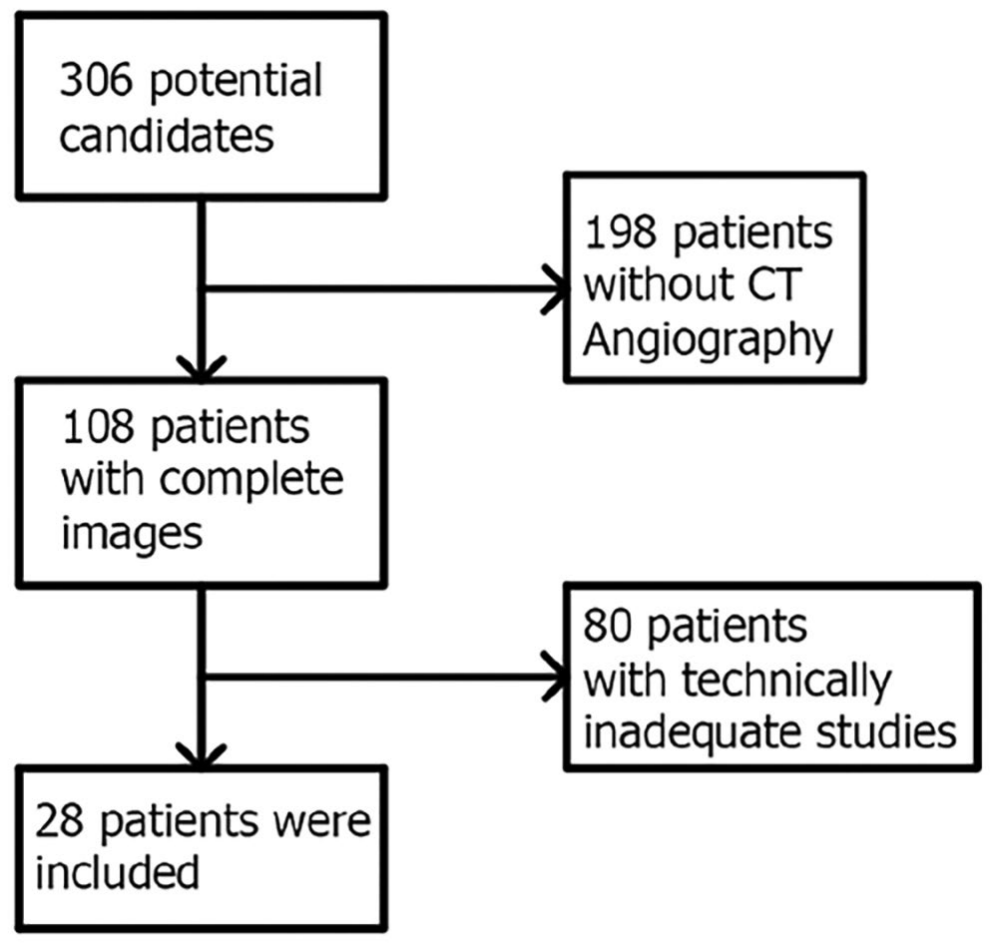
CONSORT Flow diagram

A retrospective analytical cross-sectional study was performed at Hospital Universitario del Valle in Cali, Colombia, including patients over 15 years of age who were admitted from January 2019 to December 2021 with diagnosis of civilian PBI due to firearms. The study was approved by the Health Research Ethics Committee of Universidad del Valle (Study: E034-022). The study was registered at http://openscienceframework.org (10.17605/OSF.IO/SWG43). Patients with isolated PBI or associated with extracranial wounds without airway or hemodynamic instability and evaluated with CT and angio-CT in the first 24 h after admission, were included. Patients with penetrating extracranial injuries to the lung, heart, abdomen, or visceral organs that were hemodynamically unstable or required exploratory surgery, were excluded. Subjects who died in the emergency department or before arrival, or subjects with TBI without penetration of the bullet into the skull or with a tangential trajectory, were not included. Patients with technically inadequate tomographic studies (insufficient contrast, movement or metallic artifact that make it impossible to assess the image) or with multiple potential vectors due to fragmentation of the bullet were not included (Fig. 1).

The primary outcomes include mortality and disability at 6 months. The Glasgow Outcome Scale (GOS) was used to evaluate patient´s mortality and disability at discharge and after 6 months. Secondary outcomes include clinical condition on admission, CT abnormalities, ballistics analysis, surgical treatment, length of stay and CT-angio vascular compromise.

### CT dataset

Non-enhanced head CT and CT brain angiography followed the Department of Radiology guidelines for head CT scanning. A Siemens tomograph with 128 RF was used for obtaining the images. Slices were aligned to the foramen magnum orientation with 1 mm in thickness. To compensate the 1 mm thickness of the slices, a 2 mm (1 mm) of inaccuracy for vertical structural measurements on CT scans was assumed [5, 6]. The vertical cuts were 0.6 mm of thickness in CT brain angiography and were made in a helical fashion in such a way that they were parallel to the plane of the orbitomeatal line for the exploration of the intracranial vessels after the administration of 80 milliliters of non-ionic intravenous contrast via infuser pump at 4 milliliters per second. The scan identifiers of each patient were anonymized.

### Reconstruction path in 3D slicer of CT, CTA, and MRI

The reconstruction of the projectile trajectory was made with the use of the rendering-markups format and IGT modules in 3D Slicer software (version 5.0.2) (3D Slicer (www.slicer.org*).* First, we uploaded the DICOM files and rendered the CT Volume of the study using the rendering module, varying the shift transfer function until the skull was seen. Presets CT-AAA and CT-AA2 were used to achieve this goal. Once the skull was seen, the bullet entry point was identified and labeled as “I” and the exit or resting point as “O”. Then, using the markups module, two points were created and named “I” and “O”, with respect to the coordinate system located in the dorsum sellae. These two points were used as a reference to create a line that connects them using a script code in Python language (Python (www.python.org*)).* Once the trajectory was determined, the para-axial, para-sagittal, and para-coronal angles were calculated, which were used to create the trajectory planes using the Reformat module in 3D Slicer in which the track was observed. In addition, we use metal fragments, fractured bones, and subcutaneous distortion to help determine the projectile track.

The entry site was identified in CT by the pattern of beveling of the outer and inner table, also when the fragments of the bullet remained superficial in the extracranial soft tissues of the entry site. It also usually had a smaller area than exit wounds and had an irregular stellate fracture radiating from the point of impact. The inner table often had more comminution than the outer table at the site of impact and as opposed, the outer table may have more comminuted than the inner table at the exit site. Intracranial ricochet bullets were identified by a fracture of the inner table at the point of rebound. Therefore, a bullet inside brain parenchyma with evidence of a inner table fracture in proximity to the projectile is indicative of a ricochet trajectory. The reconstruction of the ricochet trajectory involved the connection of two linear vectors, one from the entry site to the rebound site and from this internal point of inflection to the exit or resting point. All the imaging signs of entry and exit sites were correlated with the clinical examination of the patients on admission and the clinical signs that were suggestive of the entry and exit points [1, 2].

It is important to define in this section the different types of trajectories the projectile could had in relation to the skull. A penetrating trajectory only had an entrance wound, remained inside the skull and without an exit wound. A perforating trajectory had both an entry and exit wounds and a ricochet trajectory had rebounded in the internal surface of the skull [1, 2].

### Image vector analysis

**Fig. 2:**
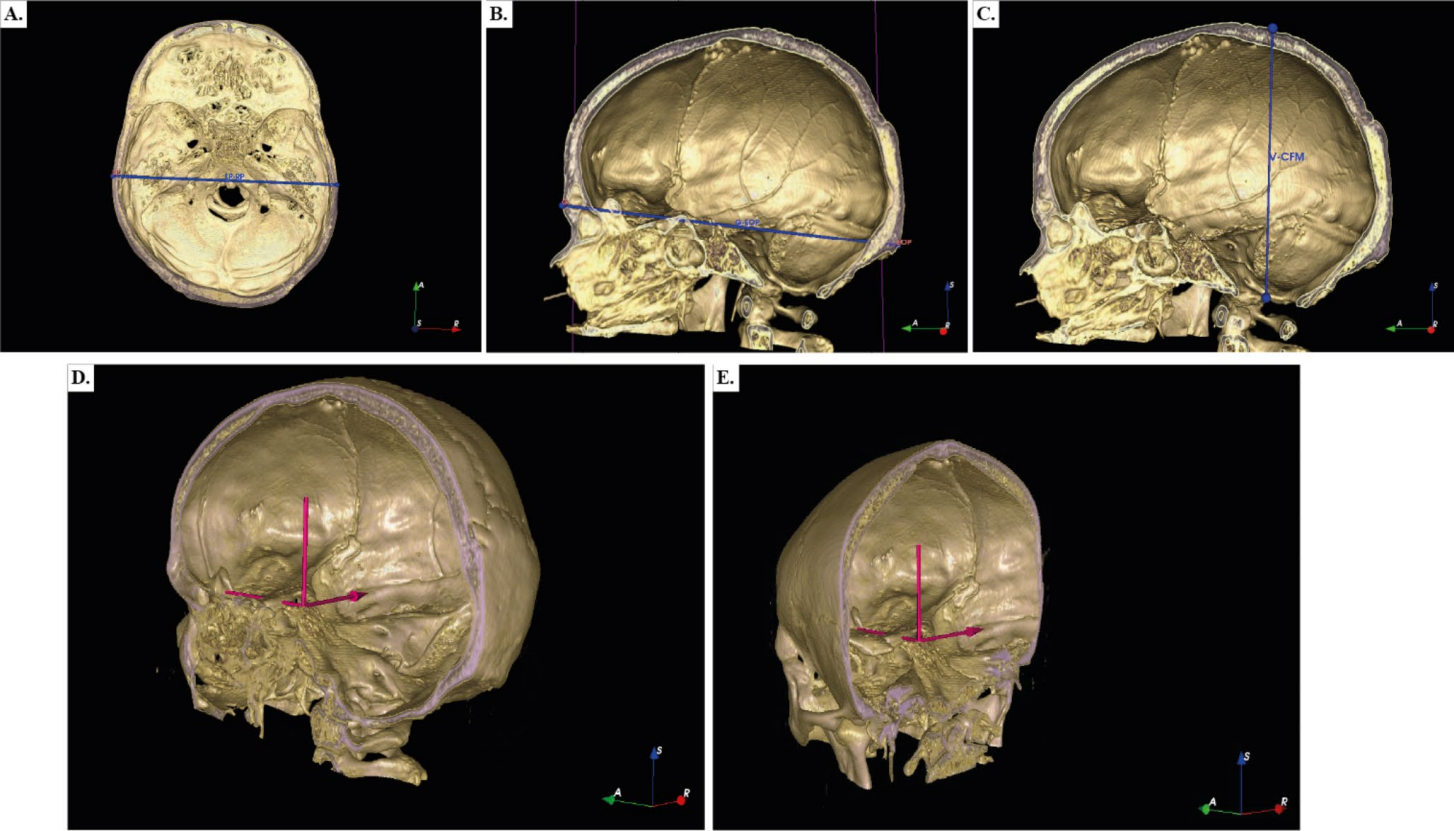
**a**. Biparietal measurement, *Xn*. **b**. Glabella-to-external occipital protuberance measurement *Yn*. **c**. The distance from the vertex to the center of the foramen magnum *Zn*. **d**. Sagittal view of the center of this cartesian system (Point *x = 0*,* y = 0*,* z =* 0). 2e. Coronal view of the center of this cartesian system (Point *x = 0*,* y = 0*,* z =* 0)

The selected images were measured in three Cartesian coordinate axes as follows: *Xn*: refers to the distance from the widest aspect of the outer left parietal bone cortex to the right parietal bone cortex of patient *n* (biparietal diameter) (Fig. 2a). *Yn*: was the distance from the external occipital protuberance to the glabella (glabella-occipital distance) (Fig. 2b). *Zn*: refers to the distance from the midpoint of the foramen magnum to the vertex (Fig. 2c). Using the IGT module, the midpoint of this Cartesian system (Point *z = 0*,* y = 0*,* x = 0*) was in the center of the superior part of the dorsum sellae (Fig. 2d and e). Vectors assessments were performed for all patients in non-enhanced CT and CT brain angiography.

The measurements were extracted and their standard deviation was computed. The anthropomorphic variation was established in an error of 1 cm according to the description of Bergland et al. [7]. Each patient’s measurements were compared with those of the patient 0 in the 3D Slicer software.

Patient 0 (baseline patient) was selected from a list of 22 possible candidates. His head size includes the average measurements of the patient’s heads in this study in terms of mean and standard deviation. Additionally, the lack of midline shift and brain edema, made him an ideal candidate to be the standard brain and cranium for comparison. This patient was a 52-year-old man with a history of structural focal epilepsy who was admitted to the hospital due to focal-onset seizures with progression to bilateral tonic-clonic seizures. In the non-contrast brain magnetic resonance imaging (MRI) he presents small areas of gliosis in the right cerebellar hemisphere and both temporal lobes. The CT brain angiography was normal. There was no healthy control for MRI and CT brain angiography in our institution that fulfill the average measurements of the patients in this study in terms of mean and standard deviation. The patient in mention was selected because represent the best of our sample. The minor structural alterations did not affect de vector analysis for TBI patients. Patient 0 had a brain non-contrast MRI in which we plot the CT findings using Cartesian coordinates, where all patients CTs measurements were replotted for further analysis of potential zones associated with adverse or favorable outcomes.

When the dimensions of patient 0 were compared with the average *Xn*,* Yn*, and *Zn* coordinates (*X* mean 137.80 mm, *Y* mean 176.10 mm and *Z* mean 141.80 mm), the difference between previous measurements and *X*_*0*_ = 141.70 mm, Y_0_ = 180.30 mm Z_0_ = 140.00 mm were well within the standard deviation *X*_SD_: 5.62 mm,*Y*_SD_:9.62 mm, and *Z*_SD_:5.88 mm.

### The measure of entry angles

**Fig. 3:**
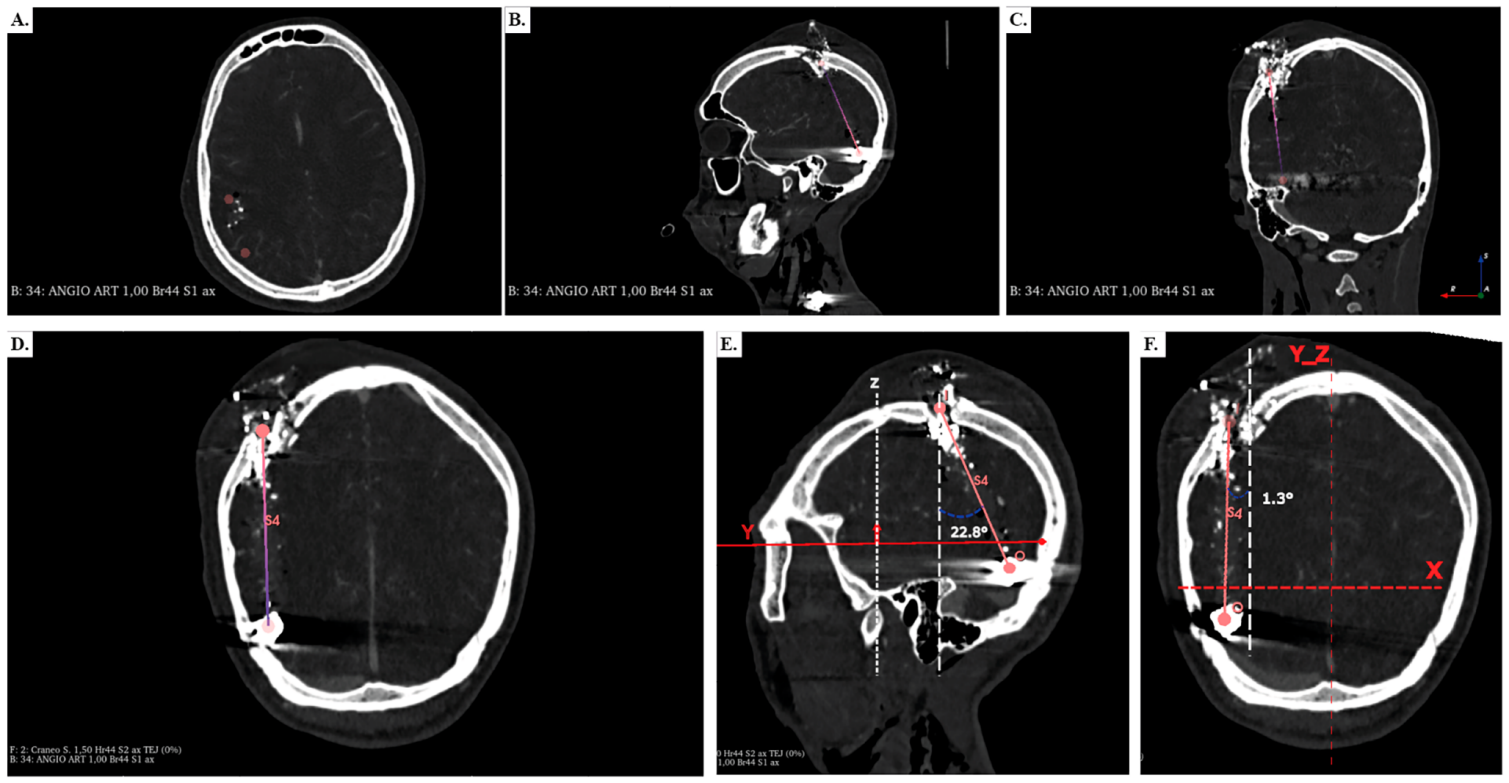
Comparison between the axial, sagittal and coronal views (classical views), regarding the para-axial, para-sagittal and para-coronal views of patient S4, a survivor, for determining resultant pathways in trajectories. **a**. Axial view. **b**. Sagittal view. **c**. Coronal view. **d**. Para-axial MPR showing trajectory 67.07° with respect P_Axi_RA (RA Axial plane), seems like the para-coronal plane. **E**. Para-sagittal CT MPR showing trajectory 22.8° toward the occipital bone, demonstrating the para-coronal orientation of the wound path. **F**. Para-coronal CT MPR showing trajectory 1.3° toward the patients Right, demonstrating the para-sagittal orientation of the wound path

The angle measure was made after obtaining the trajectory and selecting the patient 0. All the trajectories were plotted with respect to this coordinate system on the dorsum sellae. Then, using the markups module, an axial, sagittal, and coronal plane was created with respect to the coordinate system to measure the angles using the following equation sinθ = *bnbn* in python script. The *b* is the vector director of each trajectory and *n* is the normal vector of each plane. These angles are very useful in multi-planar reconstruction (MPR) [8] because they provide the ability to reconstruct complex planes that match the plane in which the trajectory is seen best (Fig. 3).

### Measures of distance to the circle of Willis

For each patient a measurement was made between the line of the trajectory and the closest node located in the circle of Willis. This measurement was made in each patient using the markups module and angio-CT, the coordinate system was placed in the dorsum sellae. All the measurements were taken and registered, and the median and standard deviation were calculated. The measurements were made using the formula *D = APvv*, where *D* is the perpendicular distance between a point P(*X*_*1*_,*X*_*1*_,*X*_*1*_) and a line with direction vector *V*(*Vx*,* Vy*,* Vz*). A is a point on the line.

### Measures of the directional cosines of bullet trajectories

A vector trajectory can be fully described by the point of entrance and exit, the length of the trajectory, and the “direction of cosines”. In the study methodology we use octans to group or classify the trajectories entry and directional cosines to describe the direction of the path. The direction of cosines are angles that were calculated with the next formulas: arc VxV, arc VyV and arc YzV. Vx, Vy and Vz are the components of the vector that defines the trajectory and V = Vx2 + Vy2 + Vz2, is the vector norm or the extent of the vector (length of the trajectory). These “direction cosines” are the angles between the vectors and each cartesian axes (X, Y, Z) and have the direction information of the trajectory. Using the Python iteration window in 3D Slicer, a script was created to calculate the “direction of cosines” in each trajectory.

### Statistical analysis

A descriptive analysis for each individual variable was conducted. We determined the proportions, central tendency, and dispersion measures. A bivariate analysis was made using the chi-square test and Fisher test to verify the strength of association between categorical variables considering a *p* < 0.05, taking mortality as the outcome variable. The analysis was performed in SPSS statistics software (IBM, Spain).

## Results

### Patient’s demographics

A total of 28 patients with PBI due to firearms who met the selection criteria were included (Fig. 1). The mean age was 26.43 ± 9.81 years. There was a male predominance of 25 patients (Table 1).

**Table 1:** Univariate and bivariate analyses testing for association between mortality and patient demographics, clinical variables, imaging characteristics and ballistics

Variables	Mortality		*P* value
	Yes *n* = 7	No *n* = 21	
**Age**,** years (mean ± SD)**	26.43 ± 9.81		
**Sex**			**1.000**
Female	1	2	
Male	6	19	
**Glasgow Coma Scale (GCS) at presentation**			**0.000**
GSC 3–7	5	2	
GCS 8–12	2	5	
GCS 13–15	0	14	
**Reactive pupil or pupils**			**0.011**
Yes	4	21	
No	3	0	
**Systolic blood pressure at admission**			**0.000**
< 100 mmHg	1	0	
100–110 mmHg	0	2	
> 110 mmHg	6	19	
Mean Arterial Pressure at admission			0.000
< 80 mmHg	1	3	
80–110 mmHg	3	17	
> 110 mmHg	3	1	
**Invasive mechanical ventilation**			**0.000**
Yes	7	8	
No	0	13	
**Isolated gunshot wound to the head**			1.000
Yes	6	16	
No	1	5	
**Intraventricular hemorrhage**			**0.000**
Yes	7	3	
No	0	18	
**Subarachnoid hemorrhage**			0.075
Yes	7	13	
No	0	8	
**CT scan**,** midline shift (mm)**			1.000
> 5 mm	1	5	
< 5 mm	6	16	
**Space occupying lesion**			**0.030**
Yes	7	11	
No	0	10	
**Gunshot wound type**			0.082
Penetrating	4	18	
Perforating	3	2	
Ricochet	0	1	
**Bihemispheric projectile trajectory**			**0.038**
Yes	3	1	
No	4	20	
**Distance to the Willis polygon**			**0.001**
< 25.3 mm	5	0	
> 25 mm	2	21	
**Glasgow Outcome Scale (GOS) 6 month**			**0.000**
GOS 1	0	7	
GOS 2–4	3	0	
GOS 5	18	0	

### Mortality and primary outcomes

A total of seven patients died (GOS = 1). Most patients (*n* = 26) presented with a single gunshot injury in the brain. One patient remained in a severe disability state (GOS = 3) at 6 month follow up and the other two patients remained in a moderate disability state (GOS = 4) at 6 month follow up. The remaining patients were at 6 months of follow up with a GOS of 5 points.

### Physical examination on admission

The Glasgow Coma Scale (GCS) at presentation was classified into three categories with the following scores: 3–7, 8–12, and 13–15. In the bivariate analysis a GCS score of 3–7 was associated with a higher mortality rate (*p* < 0.005). GCS scores of 13–15 universally survived. The status of patients with at least one reactive pupil at admission was associated with increased survival (*p* < 0.005). The absence of invasive mechanical ventilation, the presence of a systolic arterial pressure greater than 100 mmHg and a mean arterial pressure greater than 80 mmHg were associated with greater survival (Table 1).

### CT findings

Patients with space occupying lesions and intraventricular hemorrhage (IVH) were associated with increased mortality (*p* < 0.005). Pneumocephalus, midline deviation and subarachnoid hemorrhage was not significantly associated with poor outcomes (*p* > 0.005) (Table 1).

### Ballistics

In our institution, despite encountering a significant number of gunshot wound cases, comprehensive angiotomography on all patients is limited due to resource constraints. Regarding the regulation of ammunition, in Colombia, this is strictly managed by the Military Industry of Colombia (INDUMIL) under Law 1119 of 2006, which enforces high standards in the manufacture and control of ammunition. This likely explains why the missiles in our study, as detailed in our findings, were largely intact. Both the “penetrating” and “ricochet” gunshot wounds involved metal-jacketed bullets, and no mushrooming deformation was observed in the imaging. The consistency of these findings with INDUMIL’s standards suggests that the projectiles involved were jacketed, which is consistent with the lack of fragmentation seen in imaging and during surgical procedures. A total of twenty-two injuries were distributed as “penetrating” gunshot wounds, five as “perforating” type and only one as “ricochet”. All of the injuries were with low velocity projectiles. Perforating trajectory was not associated with higher mortality (*p* > 0.005). The absence of a bi-hemispheric trajectory was associated with a greater survival (*p* < 0.005) (Table 1). The entry site of the projectile and the presence of intracranial bullets was not associated with adverse outcomes. The most frequently entry sites to the skull were in frontal (*n* = 8) and temporal (*n* = 8) lobes. The angle of bullet entry was compared in the coronal plane between survivors and non-survivors. In survivors the angle of entry on the coronal plane was 36.1 ± 28.8 SD absolute degrees, with a range of 1 to 89 absolute degrees. In non-survivors the angle of entry on the coronal plane was 55.89 ± 28.74 SD absolute degrees, with a range of 9.64 to 76.13 degrees. The angle of bullet entry in non-survivors and survivors was not associated with mortality (*p*>0.005).

**Fig. 4:**
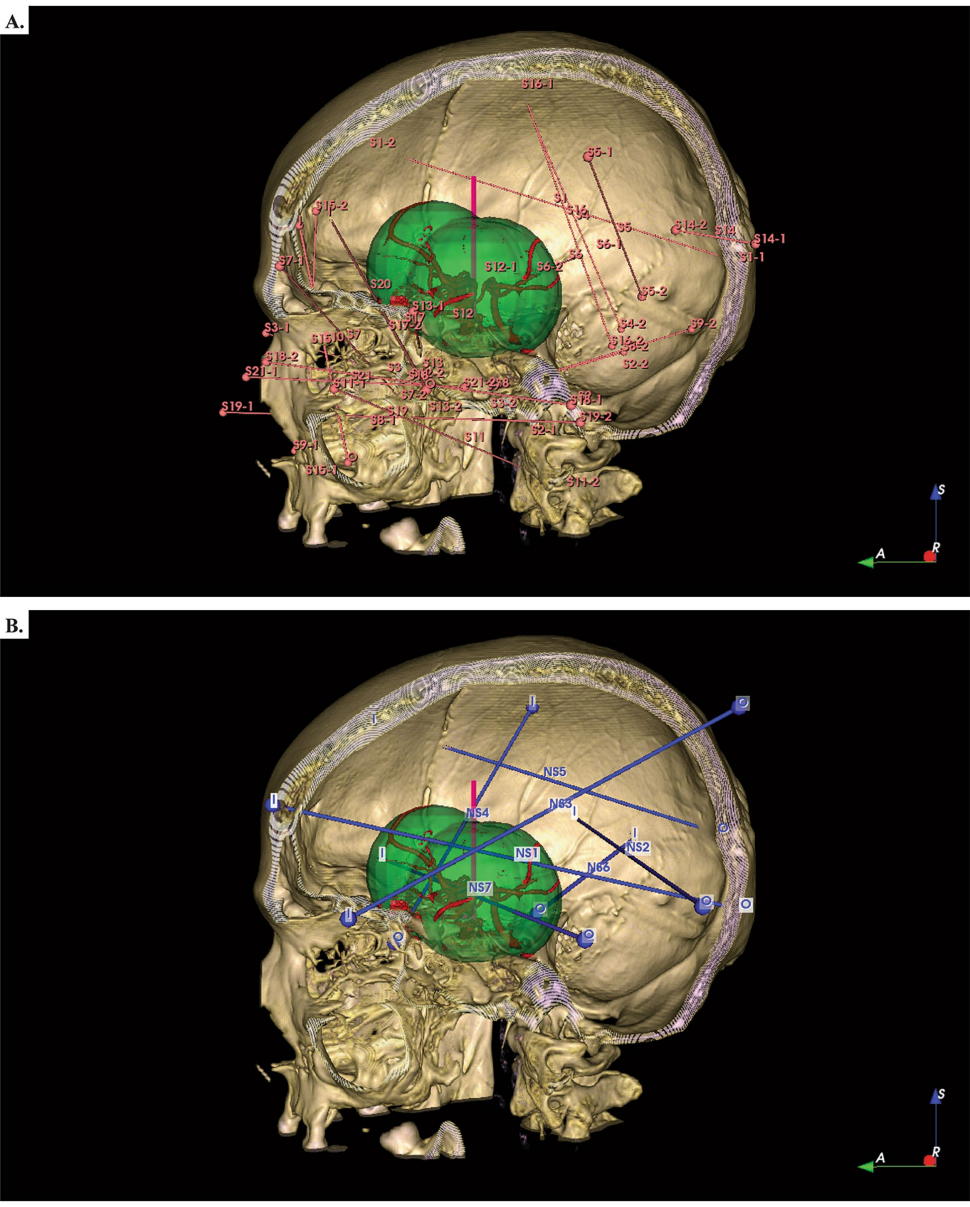
**a**. Vectorial trajectory of survivors and non-survivors. **b**. Vectorial trajectory of non-survivors

**Fig. 5:**
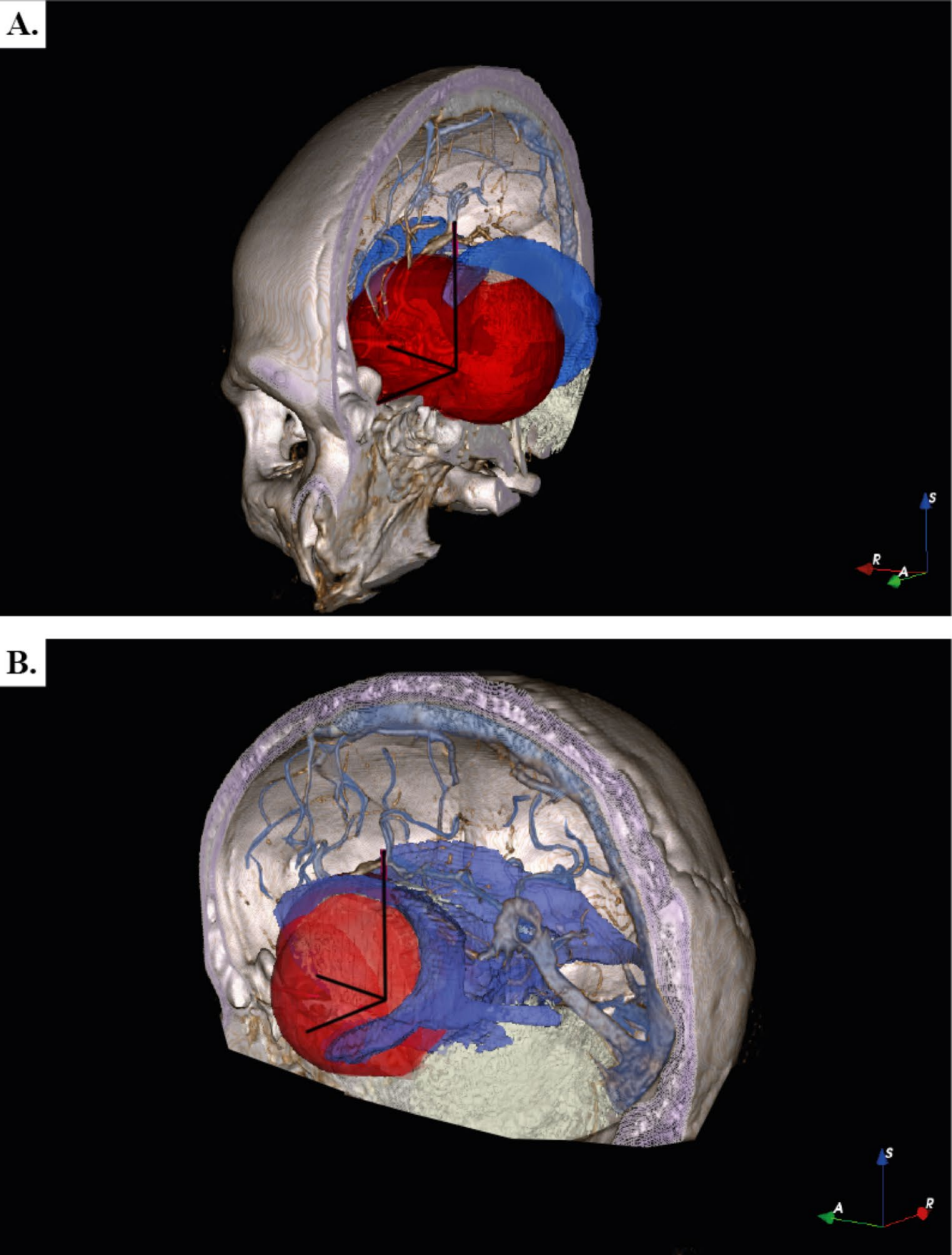
**a**. Frontal view of the “*potential lethal zone*” (in red). **b**. Lateral view of the “*potential lethal zone*” (in red). Note: Software Adobe photoshop

The survivors and non-survivors vectors were compared (Fig. 4). The vectors of the survivors exhibited a pattern in the axial, coronal, and sagittal views avoiding the ventricular system, brain stem, dorsum sellae and the circle of Willis. The vectors of 5 non-survivors tended to cross at a specific area. This area was designated as a “*potential lethal zone”*. The limits of this area include: frontal horns of the lateral ventricles as the upper limit, the brain stem as the posterior limit, the dorsum of the Turk’s saddle as the lower limit, the basal frontal area as the anterior limit and the temporal horns of the lateral ventricles as the lateral limit. This area was illustrated on patient 0’s MRI (Fig. 5). Proximity in this zone at a distance of 25.3 mm from the circle of Willis was associated with higher mortality (p < 0.005). The other two patients who died had trajectories that crossed venous structures of great relevance, one of them passing through the torcula and the other patient passing through the straight sinus.

### Surgical treatment and length of stay

The mean length of stay was 21,18 ± 29,19 SD days. Overall, seven patients died within 96 h of presentation to the hospital. Fourteen patients required surgical intervention: ten underwent cerebrospinal fluid leak correction, one had an external ventricular drain and decompressive craniectomy was performed in three patients. In our study in the group of patients who required surgical management one died, in this patient was performed a decompressive craniectomy and died in the first 70 h of presentation.

### CT angiography findings

CT angiography was completed in all 28 patients and was negative for vessel injury in twenty-four patients. In the remaining patients, findings were positive for superior sagittal sinus injury and torcula (*n* = 1), straight sinus injury (*n* = 1), pseudoaneurysm in M4 segment of left middle cerebral artery (*n* = 1) and left ophthalmic artery injury (*n* = 1).

## Discussion

### Primary outcome

Civilian PBI´s are associated with high morbidity and mortality [9–14]. In our study, seven patients died, all deaths were attributable to PBI, as we ruled out other causes of death. The outcomes of survivors at 6 months were generally favorable, with most subjects capable of independent living. The mortality rate was lower as reported in other studies [9–12] due to the several facts. First, catastrophic PBI with no CT brain angiography were excluded. Second, not all penetrating brain injuries need a vascular study for characterizing vascular relationships with PBI and therefore PBI with no CT brain angiography were excluded. Third, patients with metallic artifact with CT brain angiography that do not allow vector analysis were not included, which involve patients with multiple potential vectors due to projectiles fragmentation.

In our study, most patients were in the 3rd decade of life, which is also reported in other series [9, 15, 16]. Some studies have reported increased mortality with advancing age [15, 17]. In our study there was no relationship between sex and age with outcomes, which is reported in other studies when age is controlled by other significant factors [3].

### Physical examination on admission

The GCS is a strong predictor of survival in PBI and it has been identified as a predictor of survival [10, 18–22]. In our study, individuals at admission with a GCS score between 3 and 7 had the worst overall outcomes. We detected a clear association between pupil reactivity and favorable outcomes. Bilateral fixed and dilated pupils were associated with higher mortality. The overall patients who survived have both or at least one reactive pupil. Our findings are in line with those described in other series in the literature [19, 23, 24].

### Computed tomographic findings

Space-occupying lesions in CT scan were associated with poor outcomes, as well as IVH. The overall population who died had a space occupying lesion in CT scan. Space-occupying lesions and IVH have been associated with higher mortality rates in previous studies, which is consistent with the published literature [2, 17, 23].

### Ballistics and patient outcomes

In our study the type of injury (penetrating, perforating, ricochet) was not associated with worst outcomes. Although, a considerable number of studies have correlated perforating wounds with a higher mortality rate and poorer outcomes [2, 23]. We also did not include patients with “tangential” or “careening” types of injuries that are usually associated with favorable outcomes [3, 25]. In our study, not having a bi-hemispheric projectile trajectory was associated with a favorable outcome. Bi-hemispheric projectile trajectories have been associated with poor outcomes [2, 13–15].

All survivor’s vectors stayed away a particular area; avoiding the ventricular system, brain stem, dorsum sellae and the circle of Willis. This particular area was designated as a “potential lethal zone”. This zone was involved in 5 of 7 fatal outcomes. Proximity in this zone at 25.3 mm from the circle of Willis was associated with higher mortality. Proximity to the circle of Willis have been previously associated with arterial injuries after penetrating brain injury [26, 27], but not with higher mortality. The other two patients who died had trajectories that crossed venous structures. In previous works, Alexoupoulos et al. identified 65 lobar trajectories, reporting that bitemporal and frontal to contralateral parietal trajectories were universally fata [3]. Maragkos et al. in a recent meta-analysis identified that patients with bihemispheric, multilobar and transventricular gunshot were associated with increase mortality [19]. Kim et al. [28] described a death zone located 4 cm above the dorsum sellae, which was redefined in our study in relation to the boundaries already mentioned and the circle of Willis that was appropriately identified in the CT angiography of the patients. Therefore, our area could explain the poor outcome of previous trajectories described by those authors. The angle of bullet entry in non-survivors and survivors was compared in the coronal plane. The values were similar as in previous experiences and similarly were not associated with clinical outcomes [28].

### Surgical treatment and length of stay

In our study, the mean length of stay was 21,18 ± 29,19 SD days, which was considerably greater than other series [3]. The most common surgical intervention was cerebrospinal fluid leak correction (*n*=10).

### Angiographic findings

CT angiography was completed in all patients and was negative for vessel injury in 85.7% of individuals, which is comparable to other series [3]. In the remaining population four vascular injuries were observed (one pseudoaneurysm, one straight sinus injury, one torcula and superior sagittal sinus injury, and one left ophthalmic artery injury), similar rates have been reported also in the literature [3, 29–31].

### Limitations

The main limitations of our study are the sample size, the selection bias due to the restrictive inclusion criteria and its retrospective design. As with every retrospective design, our study is subject to selection bias and type II error. As well as survivor bias, considering the high mortality in this type of PBI. To decrease other type of biases, data was confirmed by individual files review and the clinical data and CT information was processed separately before determining the correlation of the collected information.

## Conclusions

Presenting vital signs, physical examination findings and the CT scan on admission are crucial in determining survivability following penetrating brain injury. Some brain areas had been associated with greater mortality in previous works. In our case PBI avoiding the ventricular system, brain stem, dorsum sellae and the circle of Willis were associated with more survivability. The vectors of non-survivors tended to cross at a specific area. This area was designated as a zone with a lethal worth. These findings might support the decision making for emergency management of patients with civilian PBI´s. The correlation of the GCS, the imaging pattern in CT and the ballistics trajectory analysis are a potential tool for guiding the decision making in patients with PBI, in terms of pointing which patients might not benefit of a surgical intervention due to a low GCS, signs of low prognosis in CT and a trajectory through a “potential lethal zone”. On the other hand, patients with a high GCS, absence of low prognosis signs in CT and a trajectory not going through a lethal zone; are patients with a better prognosis and a potential benefit in a surgical intervention. A better evaluation of the performance of this “potential lethal zone” in larger studies will be required. There are no new additions related to most of the CT findings associated with prognosis. Based on the findings of this study, it is recommended that further multicenter studies be initiated, involving patients with similar characteristics. Such studies would not only validate and refine the conclusions drawn here but also explore the potential for extrapolating these findings to broader populations.

## Data Availability

No datasets were generated or analysed during the current study.
